# Does aquatic exercise improve commonly reported predisposing risk factors to falls within the elderly? A systematic review

**DOI:** 10.1186/s12877-019-1065-7

**Published:** 2019-02-22

**Authors:** Eduardo Martínez-Carbonell Guillamón, Louise Burgess, Tikki Immins, Andrés Martínez-Almagro Andreo, Thomas W. Wainwright

**Affiliations:** 1 Faculty of Health Science, Catholic University of Murcia, UCAM, Av. de los Jerónimos, 135, Guadalupe, 30107 Murcia, Spain; 2 Orthopaedic Research Institute, Bournemouth University, 6th Floor, Executive Business Centre, 89 Holdenhurst Road, Bournemouth, BH8 8EB UK

**Keywords:** Elderly, Aquatic exercise, Magnitude of load, Fall prevention

## Abstract

**Background:**

According to the World Health Organization, the elderly are at the highest risk of injury or death from a fall. Age-related changes in strength, balance and flexibility are degenerative factors that may increase the risk of falling, and an aquatic training may offer a favourable environment to improve these modifiable risk factors.

**Methods:**

A systematic review was conducted to assess the potential preventative role of aquatic exercise for reducing the risk of falls in the elderly by improving predisposing risk factors. Electronic databases and reference lists of pertinent articles published between 2005 and 2018 were searched. Randomized controlled trials (RCTs) that directly or indirectly addressed the effect of aquatic exercise for the prevention of falls in healthy participants were included within the synthesis. Studies were included if they were reported between January 2005 and May 2018 within a population aged between 60 and 90 years old that were without exercise-effecting comorbidities. Data related to participant demographics, study design, methodology, interventions and outcomes was extracted by one reviewer. Methodological quality assessment was independently performed by two reviewers using the PEDro (Physiotherapy Evidence Database) scale.

**Results:**

Fourteen trials met the inclusion criteria. Exercise intervention duration and frequency varied from 2 to 24 weeks, from 2 to 3 times per week, from 40 to 90 min per session. Fall rate was not reported in any of the studies analysed. However, aquatic exercise improved key predisposing physical fitness components that are modifiable and internal risk factors for falling.

**Conclusions:**

There is limited, low-quality evidence to support the use of aquatic exercise for improving physiological components that are risk factors for falling. Although the evidence is limited, and many interventions are not well described, these results should be considered by health and exercise professionals when making evidence-based, clinical decisions regarding training programmes to reduce the risk of falling.

## Background

Fall-related injuries and deaths are serious and increasing issues for the elderly population. A fall is defined as “an event which results in a person coming to rest inadvertently on the ground or floor or other lower level, excluding intentional change in position to rest in furniture, wall or other objects” [1]. Approximately 30% of people aged over 65 years will fall at least once a year [2, 3] and 15% at least twice a year [4], and this risk increases with age. These falls may result in fracture, long-term pain, disability and functional impairments and as a consequence, an individual may suffer negative social effects, due to a lack of independence, which reduces an individual’s quality of life. In addition, falls in the elderly create an economic burden to hospitals and healthcare systems [5].

There are a multitude of risk factors for falling, and they can be internal or external and modifiable or unmodifiable. Some risk factors are directly correlated and others interact in a more complex manner [2]. Ageing is associated with anatomical and physiological changes that can lead to increased disability, frailty and a higher risk of falls [6]. Sarcopenia is the degenerative loss of muscle mass, which increases linearly with age [7]. In addition, involuntary, age-related impairments of the three sensory systems that control posture (vestibular, visual and somatosensory) can lead to falls. A study by Rubenstein [8] reviewed 16 controlled studies and found weakness, balance deficit and gait deficit to be the most important individual risk factors for falls. More specifically, a systematic review [9] found lower extremity weakness to be a clinically important and statistically significant risk factor for falls. In addition, a study by Myers et al. [10] proposed strength, flexibility, balance and reaction time to be the most modifiable internal risk factors to falls, therefore providing a rationale for exercise interventions that aim to reduce falls in the elderly.

Research shows that detection and improvement of predisposing modifiable risk factors can reduce the rate of future falls [11, 12]. Regular physical exercise has been theorised to counteract the negative physiological effects of the aging process and improve physical and mental wellbeing [12, 13]. However, barriers to exercise behaviour among older adults include a fear of falling and perceived negative affect [14] and it is therefore difficult to conclude which training prescription (for example: load control training, progression of the load during balance exercises, type of equipment, depth of water) will create an effective programme [15, 16]. Studies that have compared aquatic and land group with the same protocol, through specific functionality tests, do not report significant differences between training groups [17]. Therefore, it is possible that aquatic exercise may improve balance, strength and flexibility out of water despite the differences in the environment.

Aquatic exercise provides a low-impact and low-weight bearing environment where individuals can exercise safely. The risk of falling is eliminated, and therefore an individual can concentrate on making physical improvements. In addition to safety benefits, aquatic programs may offer an appealing alternative to repetitive, conventional exercise which may increase compliance to rehabilitation plans. There are many components of physical fitness that can be trained in an aquatic environment to help reduce the risk of falling, for example: agility, balance, co-ordination, strength, flexibility and speed. The aim of this systematic review is to evaluate the effectiveness of using an aquatic environment to reduce the most modifiable predisposing risk factors of falls within an elderly population. The type of exercise, frequency, intensity and duration of each exercise intervention will be assessed to determine if physical activity within an aquatic environment offers potential benefits to the risk of falling.

## Methods

### Protocols

This manuscript is written in accordance with the PRISMA (Preferred Reporting Items for Systematic Reviews and Meta-Analyses) statement which includes controlled and randomized studies [18, 19]. A systematic review was completed to assess the role of aquatic exercise for improving the commonly reported predisposing risk factors of falls in the elderly.

### Eligibility criteria and information sources

A computer based search was completed between January 2005 and May 2018 and the electronic databases sourced were: ISI Web of Knowledge, ProQuest, PubMed, Science Direct, SPORTDiscus, the Cochrane Central Register of Controlled Trials and the Google Academic Meta searcher. The PICO (Population, Intervention, Comparison and Outcome) framework was used to define the search strategy. The search strategy was developed to include a combination of controlled vocabulary (MeSH) and free text terms which can be found in Table 1. Reviews and commentaries were used to identify papers, but were not included within the synthesis of results. The search reviewed all available studies published between 2005 and 2018. In the mid-nineties, strong research emerged on the benefit of the aquatic environment in rehabilitation compared to the land environment. In particular, an article belong to Tovin [20] who compared the anterior cruciate ligament reconstruction water versus land exercise. However, during this time it was not shown that training in the aquatic environment could be as effective in older adults as land exercise in order to improve one’s physical condition. Until the past decade, no research groups considered that the limitation of the aquatic environment was due to the lack of control of the training load during the exercise. The year 2005 is estimated as an ideal date to begin reviewing literature as before this time, the scientific rigor of relevant studies was low as they were directed solely for therapeutic exercise such as knee injuries or hip osteoarthritis [21]. From studies where the intensity of the exercise is controlled in a systematic way through adapted material, depth, type of exercise and number of series, aquatic exercise can be better understood by healthcare professionals [22].

**Table 1:** Search Strategy

Search Strategy	
The terms “aquatic-exercise” (“water-based training”, “water exercise”, “hydrotherapy”, “exercise-aquatic) and “fall” (“fall-prevention”, “fall risk”) were combined with the terms: “older-adults”, “elderly”, “aging”, “physical-exercise”, “physical-fitness”, “balance”, “strength” and “muscle mass”.	

### Study population

The study population consisted of healthy adults aged between 60 and 80 years old.

#### Type of intervention and comparisons

To be included within the study, the articles were required to state the training methodology adopted within an aquatic environment. Studies were also included if they compared either (i) aquatic exercise and non-aquatic exercise, or (ii) aquatic exercise and aquatic exercise with different types of intervention. Studies that did not report their intervention methodology to prevent falls were excluded.

#### Type of outcomes

Assessments of physical function and/or physical performance based on fall prevention in an aquatic environment were the outcomes in the included studies. Data were extracted which related to outcomes about the intensity, volume, type of exercise, frequency and recovery.

The studies retrieved were assessed against the inclusion criteria, based on their titles. The full text of the remaining studies was then independently reviewed by two researchers (EMCG and LB). An inclusion and exclusion criteria can be found in Table 2.

**Table 2:** Eligibility criteria

Inclusion Criteria	Exclusion Criteria
Population	
Healthy adults	Adults with pathologies or comorbidities
Aged between 60 and 90	
Interventions	
Studies that directly or indirectly assess the prevention of falls in an aquatic environment	Methodology not clearly stated
Outcome Measures	
Physical function	
Physical performance based on fall prevention	
Methodology	
Randomised or non-randomised clinical trial.	Reviews, study protocols or case studies.
Publication	
Published between 2005 and 2018	Published before 2005

### Data collection process, data items and summary measures

Data were extracted by one reviewer (EMCG) using a standardized template and verified by a second reviewer (JO). Information was extracted from each included trial on: authors, year of publication, study design, population characteristics (number of participants, gender and age), description of intervention and outcome measures. Disagreements or discrepancies on data extraction were resolved by discussion.

### Methodological quality in individual studies

The methodological quality of all studies was assessed using the PEDro (Physiotherapy Evidence Database) appraisal instrument [23]. The 11 item scale is a valid and replicable measure used to critically appraise the quality of randomized controlled trials. Each study is scored out of ten, with a threshold of seven (or over) for a study to be considered high quality (item one on the scale indicates external validity).

## Results

**Fig. 1:**
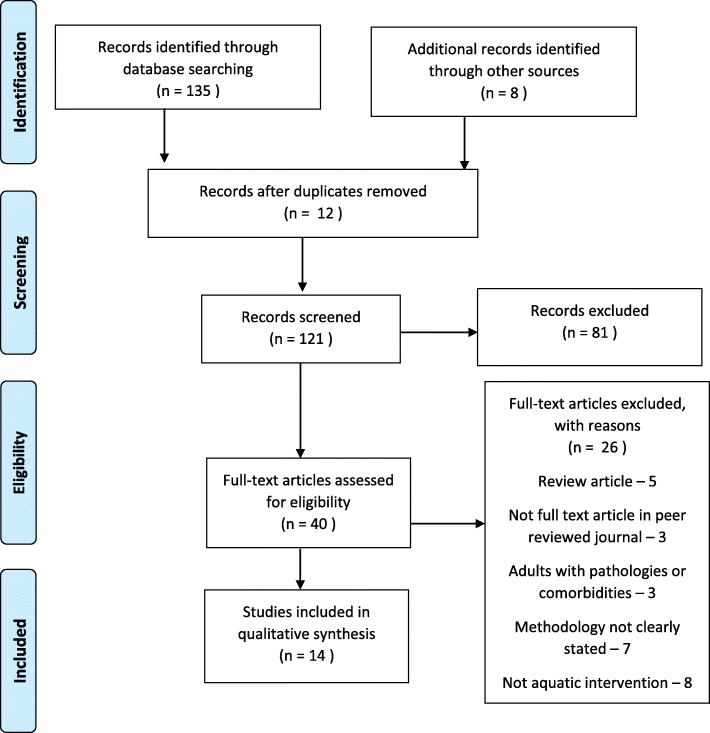
Flow chart illustrating each phase of study selection

A summary of the selection process is schematized in Fig. 1. The literature search yielded a total of 143 articles, of which 12 were duplicates and 81 were immediately excluded after reviewing the title, as they did not fit the inclusion criteria. A total of 40 full-text articles were reviewed. Thirty-two articles could be obtained through the search engine of the Catholic University of Murcia and its corresponding subscriptions to certain journals. The eight remaining articles were obtained on free access web pages or through an interlibrary loan process. Following a full text review (EMCG and TI), fourteen studies were included in the synthesis of results.

Twelve of the sourced studies were randomized controlled trials (RCTs) [16, 24–34] and two studies adopted a quasi-experimental design [35, 36]. Nine studies specifically evaluated the impact of aquatic training on improving strength [16, 25, 27, 29–32, 35, 36], nine studies assessed balance following aquatic training [24, 26, 28–30, 32, 33, 35, 36], and four studies compared a variety of physical fitness components, including agility, endurance, flexibility and speed [26, 27, 35, 36].

### Measuring instruments

The flexibility variable was recorded by the chair sit-and-reach and the back scratch test in one study [26, 30] whilst another study used the functional tests of Johnson and Nelson [35, 36]. The remaining studies utilised the sit and reach test [25, 27, 29].

Dynamic balance was measured using the berg balance scale [33], the timed up and go test [24, 25, 27, 28, 35], the 8 ft up and go test [29, 30, 34] and the walking in circle test [36]. Static balance was recorded with the Sharpened Romberg test [24, 28, 35] with both open and closed eyes, it was also registered by a Biodex Balance Stability System [32] and using a force platform where the subjects kept their arms on their hips to maintain position [36].

The variable of strength was predominantly recorded for upper limbs using a hand dynamometer [25, 27, 30, 32]. The maximal dynamic strength was evaluated using the 1-repetition maximum (1RM) bench press [16] bilateral elbow flexion [16, 26, 36] and bilateral knee flexion/extension [16]. The muscular endurance was evaluated during a bilateral knee extension and flexion with a load equivalent to 60% of 1RM maintaining a maximum possible number of repetitions [16]. An isokinetic machine was also used for lower limbs. In this case a knee flexion-extension is performed at speeds of 60°/s,120°/s [26]. In addition, a functional test like the 30 s sit-and-stand-up were used by a large number of researchers [26, 29, 31, 36].

Flexibility was mainly measured by the sit-and-reach test for the hamstrings muscles and lower back [25–27, 29, 30] and the back-scratch test was used to assess the general shoulder range of motion [26, 30].

### Study population

When divided into their respective experimental groups, nine of the studies sourced had sample sizes of between 13 and 17 subjects [16, 24–27, 30–33, 35]. The remaining studies tested between 20 and 66 subjects [28, 29, 34, 36]. Four studies assessed both male and female participants [27–30], four studies only included women [16, 25, 31, 36] and three studies only assessed male participants [24, 32, 35]. The age range of the study populations was generally small, however in four studies the range was 15 years [16, 25, 29, 35] and in two studies the range was over 24 years [33, 36].

### Intervention variables

The temperature of the water varied between 28 °C and 32 °C within the majority of studies, [16, 25–27, 29, 34–36] with the exception of four studies, [24, 28, 30, 31] where the temperature was over 32 °C and two studies which do not specify the water temperature [32, 33]. The depth of the water in most of the interventions was from between waist to the midline of the chest [24, 26, 28] and at the level of the xiphoid process [16, 25, 29–31, 33–36] except in two studies [30, 32] where the depth reached 1.80 m. The shorter studies had a duration of two weeks [28] and six weeks [24, 30]. Most interventions were implemented for eight to 12 weeks [16, 26, 27, 29, 31–35]. However, one study completed a 16 weeks intervention [36], and the longest intervention had a duration of 24 weeks [25]. The frequency of the intervention was generally between two sessions [16, 24, 30, 31, 33, 35, 36] and three sessions a week [25–27, 29, 32, 34] although there is a study with a frequency of 5 days a week for 2 weeks during the intervention [28]. In terms of duration, most of the interventions were 60 min long, [25, 26, 28–30, 32, 35, 36] however, one study had a duration of 90 min [28], another of 40 min [34] and four studies did not specify the duration of the intervention [17, 25, 32, 35].

In some cases, the intensity of training was described by referring to the Borg Rate of Perceived Exertion Scale, the same Borg scale which ranges from six to 20 points. A value of one represents a very light exercise and 20 a serious physical exertion [37]. Moderate to high intensity was mentioned in ten of the included results [16, 25–27, 29–31, 34–36] and exercise intensity was not systematically recorded in the remaining studies [24, 28, 32, 33].

### Physiological improvements

The control group, absent from any intervention, did not obtain any significant difference at the end of the study [25, 26, 28, 30–36]. However, the aquatic intervention group obtained improvements (*p* ≤ 0.05) at the end of the study in the variable of flexibility [25–27, 29, 30, 36], balance [24, 26, 28, 32, 35, 36] and strength [16, 25–27, 29–32, 36].

Regarding the four studies that include a third exercise programme in a land environment, two of them did not find differences between the aquatic and land groups after the intervention, in variables of flexibility, balance and strength [24, 28]. Nevertheless, the aquatic group obtained greater improvement over the terrestrial group in the variable of balance in one study [30]. Another study [27] compared two aquatic groups with different types of intervention: resistance material and without any material. The material group presented better results in gait speed, however, in variables of strength, mobility and flexibility, no significant differences were found between groups.

### Resistance material

The studies did not specify what kind of materials they used except in one case [27] where the aquatic group used newly made water-resistance equipment (power leg and power hand DESCENTE Co., Ltd., Japan) and another study [36] where the aquatic group progressed at their own rate by adding surface area equipment (Aqua Flex paddles by MIZUNO Corporation, Osaka, Japan) and by opening the webbing of the Aqua Mitt gloves to increase surface area to reach the goal RPE.

### Type of exercise

The studies that introduce flexibility training do not indicate specific exercises that are performed [26, 27, 29]. Only five studies indicate the static and dynamic balancing exercises which they use [26, 28, 33, 35, 36]. When looking at strength training there is a general focus on large muscle groups from the upper or lower limbs [16, 24, 26, 27, 31, 32, 34]. Only two studies [31, 36] mention the execution of exercises that resemble activities of daily living (ADL). A schematic compression can be viewed in Table 3.

**Table 3:** Studies and interventions (including assessments, results and limitations found in each study for clinicians and researchers)

Study	Objective	Population	Intervention group							
			Depth & Tª	Magnitude of load				Assessment	Outcome	Limitations of the study
Silva et al., 2018 [31]	To investigate the effects of two water programs on functional capacity and quality of life of elderly women.	Forty-one elderly female (65 ± 4 years) were divided into aerobic training group (*n* = 13), combined training (*n* = 11) and CG (*n* = 9).	Depth: Between the xiphoid process and shoulders. Tª: 33 °C	AG(I): Aerobic intervention; AG(II): Combined Intervention; CG: No intervention				Strength: 30-s chair-stand.	The 30-s chair-stand test resulted in an increase of 32 ± 11%, 24 ± 14% and 20 ± 9% for AG1 (I), AG (II) and CG, respectively.	The absence of a water-based resistance training group to compare the adaptation with water-based groups. The lack of control of the exercise program intensity and the frequency of elderly women in CG.
				Volume			Specific intensity control			
				W	F	D	Maximum effort			
							1–6 sets of 30″-5′/30″-2′ rest; AT: 85–110%			
				12	2	N/A				
				Type of exercise						
				Balance		Not included				
				Strength		Combined exercise UL and LL				
				Flexibility		General - Does not specify exercises				
Reichert et al., 2018 [16]	To compare the effects of 1 × 30″, 3 × 10″, and 1 × 10″ water resistance training on muscle strength and functional capacity in older women.	Thirty-six healthy women (60–75 years) AG (I): *n* = 12, AG (II): n = 13 and AG (III): n = 11 were divided into three different aquatic training.	Depth: Between the xiphoid process and shoulders. Tª: 31 °C	AG (I): 1 × 30″ AG (II): 1 × 10″; AG (III): 3 × 10”				Strength: Maximal dynamic strength (1-RM) Muscular endurance with a load of 60%RM.	The main finding was that the three groups strategies performed twice a week induced similar relevant improvements in maximal strength, muscular endurance, and functional capacity. However, only the AG (I) 1 × 30s and the AG (II) 1 × 10s showed increased maximal strength in the bench press exercise.	The absence of an evaluation of the explosive strength, which is also important for the elderly population, as it is related to the capacity to perform daily life activities and risk of falls.
				Volume			Specific intensity control			
				W	F	D	RPE: 19			
				12	2	N/A	Maximum velocity 1–3 sets of 30″/ 2′ rest.			
				Type of exercise						
				Balance		Not included				
				Strength		Knee and elbow flexion/extension Unilateral hip adduction and abduction				
				Flexibility		Not included				
Seyedjafari et al., 2017 [32]	To investigate the effect of deep aquatic exercises on lower body strength and balance	Thirty elderly men over 65 were divided into AG (*n* = 15) and CG (n = 15).	Depth: Over 2 m. Tª: Does not appear.	AG: Intervention; CG: No intervention				Balance: Biodex Balance System. Strength: Hand-Held Dynamometer	All variables including lower body strength, static balance was significantly improved (*p* < 0.001) in experimental group after aquatic exercises program.	The method of participant selection was by volunteer and therefore subjects were not randomly selected. This could affect the generalizability of the outcomes.
				Volume			Specific intensity control			
				W	F	D	Not included			
				8	3	60’				
				Type of exercise						
				Balance		Float exercises				
				Strength		Hip abduction/adduction/flexion/extension				
				Flexibility		General - Does not specify exercises				
Bento et al., 2015 [34]	To evaluate the effects of a water-based exercise program on static and dynamic balance.	Sixty-five independent participants (over 60 years old) were divided in AG (*n* = 20) and CG (*n* = 16).	Depth: Xiphoid process. Tª: 28–30 °C.	AG: Intervention; CG: No intervention				Balance: Displacement of the center of pressure in a quiet standing position and 8-Foot Up-and Go.	No differences were found in the center of pressure variables; however, the AG group showed better performance in the 8 Foot Up-and-Go Test after training (5.61–0.76 vs. 5.18–0.42; *p* < 0.01).	The physical conditioning of the participants at baseline revealed a good status, which may have reduced the magnitude of the training effects.
				Volume			Specific intensity control			
				W	F	D	RPE: 12–16			
				12	3	N/A	HRR: 40 to 60% 3 sets × 40″ / 20″ rest			
				Type of exercice						
				Balance		Not included				
				Strength		Hip/knee flexion/extension and dorsal and plantar flexion of the ankle				
				Flexibility		General - Does not specify exercises				
Kim y O’Sullivan, 2013 [28]	To examine the effects of aquatic exercise on biomechanical and physiological elements influencing gait.	Healthy women (70–78 years) bone mineral density score up −1. AG: *n* = 8 CG: *n* = 7	Depth: Between waist and chest. Tª: 28 ± 1 °C.	AG: Intervention; CG: No intervention				Balance: Gait observations, kinematics at 60 Hz were recorded by 8 cameras, and kinetics on by 2 force platforms. Strength: 30″ chair stand, arm curl. Flexibility: Chair sit-and-reach Back scratch	AG obtain reductions (p < 0.05) in body weight, and body fat mass, and stride time. Significant increases (p > 0.05) in leg strength corresponded to the maximum joint moment of the landing leg, getting better the ability for recovery of balance after any perturbation.	High dropout rate. The evaluation of the balance is not clear and the study does not indicate clearly the load magnitude of training in AG. Large sample difference between intervention group and control group.
				Volume			Specific intensity control			
				W	F	D	RPE: 7–11			
				12	3	60’				
				Type of exercise						
				Balance		Static and dynamic				
				Strength		General Exercise for UL and DL				
				Flexibility		General - Does not specify exercises				
Sanders, Takeshima, Rogers, Colado y Borreani, 2013 [36]	To evaluate the aquatic environment in the improvement of the ADL in women over 60 years.	Women (60–89 years), sedentary, independent and confirmed by a physician their healthy situation. AG: *n* = 48 CG: *n* = 18	Depth: Xiphoid process. Tª: 28–29 °C.	AG: Intervention; CG: No intervention				Balance: One leg with eyes open and maintain balance while they walk in a circle. Strength: Sit-to- stand and arm curl. Flexibility: Sit-and-reach	AG obtained improvements with respect to CG in balance, flexibility, strength and agility (p < 0.05).	Long difference in sample size between intervention group and control group. Short duration of intervention and important difference of age between participants. Absence of specificity of training load. The type of exercise in the AG is not specified.
				Volume			Specific intensity control			
				W	F	D	Progressive to Moderate;			
				16	3	25–45’	2 sets × 10 rep. /15″ rest.			
				Type of exercise						
				Balance		Static and dynamic				
				Strength		Combined exercise for UL and DL.				
				Flexibility		Does not specify exercises				
Bergamin et al., 2013 [30]	To evaluate the effects of aquatic exercise on the strength and functionality of the elderly.	Healthy elderly (70–76 years) were divided into aquatic group (n = 17), land group (n = 17) and control group (*n* = 19)	Depth: 1.30–1.80 m. Tª: 36 °C.	AG and LG: intervention; CG: no intervention				Balance: 8-ft up-and go Strength: Dynamometry for hand-grip and isometric knee flexion-extension Flexibility: Back-scratch test and sit-and-reach.	It has found significant improvement (p < 0.05) in flexibility, mobility and balance, obtaining AG a greater improvement dynamic balance a loss weight (P < 0.05)	Low sample size. Lack of specificity of the height of the water during the development of exercises.
				Volume			Specific intensity control			
				W	F	D	RPE: 13–16			
				6	2	60’	HRmáx: 55–65% 3 sets × 1′ / 30″ rest			
				Type of exercise						
				Endurance		Combined exercise for UL and DL				
				Strength		Combined exercise for UL and DL				
				Flexibility		Combined exercise of UL and DL maintaining movement 90″.				
Elbar et al., 2013 [33]	To evaluate a perturbation programme of balance targeted compensatory and voluntary stepping to improve speed of stepping.	34 healthy volunteers (64–88 years) was divided in two groups n = 17, respectively.	Depth: Xiphoid process. Tª: Does not appear.	AG: Intervention; CG: No intervention				Balance: Fall Efficacy Scale. Folstein Mini-Mental State Examination. Voluntary Step Execution Test Stabilogram-Diffusion Alysis. Berg Balance Scale. Get-up-and-go.	A significant interaction effect between group and time was found for the step execution, due to improvement in initiation phase and swing phase durations in the AG. Also, significant improvement in postural stability in eyes open and closed conditions is noted.	No benefits for performance aspects of balance control. Although this lack of improvement could be due to ceiling effects, it may reflect the specificity of training principle and the need for therapists to tailor balance training programs to target specific aspects.
				Volume			Specific intensity control			
				W	F	D	Progressive levels of balance intensity			
				12	2	40’				
				Type of exercise						
				Balance		Level 1: Standing - External support Level 2: Standing - No external support Level 3: Single leg – No external support Level 4: Gait exercise – No support Level 5: Perturbation and water turbulence				
				Strength		Not included				
				Flexibility		Not included				
Javaheri, Rahimi, Rashidlamir y Alikhajeh, 2012 [24]	To compare aquatic and terrestrial environment in the improvement of the static and dynamic balance for elderly.	Thirty older adults (63–70 years); independents in the daily activities was divided in two groups (n = 15)	Depth: Between waist and chest. Tª: 33 °C.	AG: Aquatic intervention; LG: Land intervention				Balance: Sharpened Romberg (open eyes and closed eyes) and Timed up & Go.	After the intervention, improvements were found in AG and LG in balance (*p* < 0.05). It was not found differences between groups AG and LG (*p* > 0.05).	Low sample size and only male. Absence of specificity in both groups.
				Volume			Specific intensity control			
				W	F	D	Progressive levels of balance intensity 1 set × 15 rep.			
				6	2	N/A				
				Type of exercise						
				Balance		Combination of displacement				
				Strength		Marching in place. Hip flexion/extension. Hip abduction/adduction. Toe raises/heel raises. Shallow knee bends. Sit to stand from chair in land group. Sit to stand from pool shelf in aquatic group.				
				Flexibility		Not included				
Alikhajeh, Moghaddam & Moghaddam, 2012 [35]	To evaluate the effect of hydrotherapy on the static and dynamic balance.	Twenty-eight healthy sedentary elderly men (64–79 years; 14 in the experimental group and 14 in the CG).	Depth: Xiphoid process. Tª: 28–29 ° C.	AG: Intervention; CG: No intervention				Balance: Sharpened Romberg test and Timed Up & Go.	AG obtained improvements with respect to CG in balance, flexibility, strength and agility (p < 0.05).	Low sample size, short duration of intervention and important difference of age between participants. Absence of specificity of training load. The type of exercise in the AG is not specified.
							Specific intensity control			
				W	F	D	Progressive to Moderate; 2 set × 10 rep. / 1′ rest			
				8	2	60’				
				Type of exercise						
				Balance		Static and dynamic				
				Strength		Combined exercise for UL and DL.				
				Flexibility		Does not specify exercises				
Bento, Gleber Pereira y Rodacki, 2012 [29]	To analyze the effects of aquatic exercise on improving LL strength and older functionality.	Elderly (60–75 years) able to walk and perform their daily tasks independently. AG: *n* = 24 CG: n = 14	Depth: Xiphoid process. Tª: 28–30 °C.	AG: Intervention; CG: No intervention				Balance: 8-ft up-and go Strength: 30″ chair-stand test. MVIC: peak torque and rate of torque development tests Flexibility: Sit-and-reach	AG obtained improvement in strength of UL (p < 0.05) and functionality (p < 0.05).	Importance difference between intervention group and control group. Absence of specificity of training load.
				Volume			Specific intensity control			
				W	F	D	HRmáx: 40–60% RPE: 12–16 40″ moderate speed / rest 20”			
				12	3	60				
				Type of exercise						
				Endurance		Displacement				
				Strength		Combined exercise for UL and DL				
				Flexibility		General - Does not specify exercises				
Walia y Shefali, 2012 [28]	Comparing aquatic and land exercise in the improvement of balance in elderly.	Healthy asymptomatic elderly (65–71 years) who were divided in a land group (*n* = 30) and an aquatic group (n = 30).	Depth: Between waist and chest. Tª: 35° ± 2 ° C.	AG: Aquatic intervention; LG: Land intervention				Balance: Sharpened Romberg (open eyes and closed eyes) and Timed up & Go.	After the intervention, improvements were found in AG and LG in balance (p < 0.05). It was not found differences between groups AG and LG (p > 0.05).	Small sample size and shorter duration are the major limitations of the study. Lack of information about the periodization of the intervention in AG. Absence of specificity of training load.
				Volume			Specific intensity control			
				W	F	D	Not included			
				2	5	60’				
				Type of exercise						
				Balance		Alternate and dynamic movements of UL and DL				
				Strength		Not included				
				Flexibility		Not included				
Katsura et al., 2010 [27]	To assess the efficacy of aquatic exercise in older adults using resistance material.	Twenty healthy elderly individuals (68–75 years) who did not exercise regularly. A resistance equipment group (n = 12) and a non-resistance equipment group (n = 8).	Depth: Does not appear. Tª: 30–32 °C	AG (I): Intervention with resistance equipment AG (II): Intervention without resistance equipment				Balance: Timed Up & Go Strength: Hand held dynamometer Flexibility: Sit and reach	AG (I) and AG (II): improvements (p < 0.05) in flexibility, strength (plantar flexion) and in static equilibrium. AG (I) obtained greater improvements (*p* < 0.05) than GA (II).	Low sample size. Under sample size and long duration of the training programme. Too much gender difference in the sample (women 16, men 4). Absence of control group. Absence of load magnitude for the intervention group.
				Volume			Specific intensity control			
				W	F	D	RPE: Moderately strong			
				8	3	90’				
				Endurance		Displacements				
				Strength		UL and abdomen				
				Flexibility		General - Does not specify exercises				
Tsourlou, Benik, Konstantina, Dipla y Kellis, 2006 [25]	To determine the effectiveness of an aquatic training programme in healthy women over age 60.	Twenty two healthy elderly (60–75 years; AG: n = 12; CG: *n* = 10) without any medical contradiction.	Depth: From the xiphoid process to the axillary region. Tª: 30 °C.	AG: intervention; CG: no intervention				Balance: Timed Up & Go Strength: Isometric test of knee’s flexo-extension; Isometric wrist grip. 3 RM in machine for UL and DL. Flexibility: Sit and reach.	AG obtained improvement in pre/post-test in balance, strength and flexibility (*p* < 0.0125). AG obtained improvement (p < 0.0125) with respect to CG in strength and balance.	Low sample size.
				Volume			Specific intensity control			
				W	F	D	EV, MA, Rhythm of movement. ± 60 b / min 2–3 set × 12–15 rep./20–30″ rest			
				24	3	60				
				Type of exercise						
				Balance		Not included				
				Strength		15–25 min of global exercise for UL and LL				
				Flexibility		Not included				

*CG* Control group, *AG* Aquatic group, *LG* Land group; (I), First group; (II), Second group; (III), Third group, *ADL* Activity daily life, *Tª* Temperature, *N/A* Not included in the text, *W* Weeks of intervention, *F* Frequency, *D* Total duration of season, *RPE* Rating of perceived exertion Borg Scale 6–20, *EV* Execution velocity, *MA* Materials of resistance, *HRmáx* Maximum heart rate, *AT* Anaerobic threshold, *HRR* Heart-rate reserve, *UL* Upper limb, *LL* Lower limb, *RM* Repetition maximum, *MVIC* Maximal voluntary isometric contraction

## Discussion

There is evidence to support the use of aquatic exercise in order to modify variables related to the risk of fall in the elderly. Aquatic exercise may ameliorate the negative physiological effects of aging which are predisposing, modifiable risk factors of a fall. Improving these risk factors is likely to create an overall benefit to rate of falls, and thus aquatic exercise should be considered as a training method for those most at risk of falling. However, the relationship between aquatic exercise and fall prevention is not directly compared within the studies sourced.

### Balance

Studies which assessed balance within their training programmes found significant improvements (*p* ≤ 0.05) following the intervention [24, 28, 32, 34–36] with the exception of two studies [26, 33]. The lack of positive results in balance training may be due to the lack of scientific rigor and the specificity of training. In this review, the exercises used most for balance training are alternating movements of the upper and lower limb [24, 26, 28, 33, 35, 36] however, their training variables such as the control of intensity or the specific exercises are not clearly reported in the methodology. Balance is considered the most important variable during the design of a fall prevention training programme [38], and consequently the specificity of the exercises and the control of training should appear in order to reproduce the results obtained in the studies. Therefore, certain criteria or principles that support the decisions made during the fall prevention program should be considered during the design of protocols to improve balance in older people. An example are those established by two experts [8, 38]: the reduction of the support base, the movement of the centre of gravity, the mobilization of the limbs and the reduction of the visual field [8, 38]. It has been recommended that due to the aquatic turbulence during the aquatic exercise, muscle are activated continuously to stabilize the position of the individual [16]. However, the depth of the water and velocity of exercise should be controlled in order to modify muscle activation during balance exercises [39].

One study [36] demonstrated that in a group of 66 subjects (60–89 years), during a 16-week programme which included strength training, in an elderly population it is possible to achieve a better static balance in the elderly. A similar study [24] evaluated the static and dynamic balance in two groups of 15 subjects (63–70 years) during two equally designed training programmes in both a land and aquatic environment. At six weeks, both groups achieved significant improvements in static and dynamic balance (*p* < 0.05) when compared to pre-test scores. Similarly, a significant gain (p < 0.05) was found for the Berg Balance Score in adults older than 68 years old with no significant difference from one environment to another (*p* > 0.05).

Although the studies reviewed did not find significant differences between land and water training, the results suggest that an aquatic environment offers a safe environment where the risk of falling is eliminated. It should, however, be noted that there is evidence to highlight the insufficiency of the Berg Balance Scale for predicting falls [40] and the Timed Up and Go Test is instead recommended by the U.S. Centres for Disease Control and Prevention as an easy to administer clinical test to evaluate a senior citizen’s fall risk [41].

One study assessed the effect of aquatic training on agility [20]. The authors found significant improvements in the intervention group receiving an aquatic exercise programme in comparison to the control group (*p* < 0.05). There is evidence that agility training programmes can reduce the risk of falls [42], largely due to improved balance, which has been examined as a variable for improving mobility in older adults. A study comparing fall prevention programmes in older adults [43] found that visual training had the greatest improvement on obstacle course performance (22%) following a 12-week training program. In addition, agility has been found to reduce the risk of falling through step training [44], and in multifactorial programs which focus on other variables such as balance and strength [45].

### Strength

Aquatic interventions that trained large muscle groups of the upper and lower limbs found significant improvements in strength from pre-test to post-test (*p* < 0.05) [16, 25–27, 29–32, 36]. Although the methodologies of these protocols vary, all results indicate that an aquatic environment is beneficial for promoting muscle hypertrophy. Strength training cause changes in the nervous system through neuromuscular activation, resulting in improved muscular power and delayed onset of sarcopenia which may decrease the risk of falls [46]. Evidence for the effectiveness of lower limb strengthening exercises for the prevention of falls in elderly subjects has been previously reported [47–49] and as lower extremity weakness is a clinically significant risk factor of a fall. The design of aquatic exercise programmes should focus on the lower limbs, respecting the principle of training specificity. Lower limb weakness in older adults is considered an important influencing factor of falls and its preservation promotes the maintenance of independence. More specifically, the maximum voluntary isometric strength and the rate of force generation of the hip abductors are considered a useful measure to distinguish between older adult fallers and non-fallers [50]. However, several studies use the handgrip dynamometer as a measure for strength in the elderly [25, 27, 32]. Although this outcome measure does not specifically assess lower limb strength, a recent study developed by the Cooperative Health Research in the Region of Augsburg [51] with 808 individuals (age ≥ 65 years) found a trend toward an indirect effect of grip strength on balance problems (*p* value = .043). In addition, handgrip strength has been estimated by other studies as an Indicator of Health-Related Quality of Life in Old Age and fall prevention [52, 53].

### Flexibility

Axial stiffness can occur through ageing and is a loss of flexibility and elasticity in the neck and trunk that causes the imbalance of the posture, thus reducing balance and increasing the chance of falling. The ACSM (American College of Sports Medicine) [47] highlights the importance of flexibility training during aging for fall prevention; however often it remains neglected in training programmes.

Within this review, six studies assessed flexibility as an outcome following aquatic training [23, 25, 27, 29, 30, 36]. In all cases, flexibility was seen to improve significantly from pre-test to post-test (*p* ≤ 0.05), however the specific effect on each muscle group was not clearly described. Regarding the principle of specificity and the relationship of flexibility with the risk of falls, the findings indicate that the rigidity in the pelvic muscles causes an alteration in the gait of the elderly, which will contribute to an increased risk of falls [54]. In this case, a specific program of hip flexor stretches is needed, which may reverse variables that increase the risk of falls in the elderly [55].

### Duration

Six studies [16, 26, 29, 31, 33, 34] agree with others found in the literature [56–58] that a training programme that seeks to allow for significant changes in balance, strength or flexibility to prevent falls in older adults should be a minimum duration of 12 weeks. To make a positive impact, the duration of the session should be around 60 min and two to three times a week [16, 26, 29, 31–34], as recommended by similar studies [48, 57, 59, 60]. The ACSM expresses an optimum frequency of three times a week at a moderate-to-high intensity to facilitate fall prevention training in the elderly [47]. This exercise prescription allows for physical recovery that induces physiological adaptations (48–72 h between sessions) [61]. However, studies with less frequency and duration also found significant improvements in balance, strength and flexibility tests [24, 27, 30, 32, 35, 36], after their respective interventions. These results indicate that the intensity of a programme will be a greater modifier of effect than the volume of training to reach physiological adaptations in the elderly and to improve functional capacity [16, 62, 63]. Therefore, it seems likely that frequency, duration and intensity of exercises, including those related to strength, balance, and flexibility are relevant training principles for reducing the risk of falls in the elderly.

### Intensity

The intensity of an exercise programme is an important factor for determining the physiological stress and subsequent adaption an individual can experience through training. If the frequency and intensity of the training programme is too high, muscular damage may occur and this can negatively affect adherence to programmes [64]. The Borg Scale is a valid and simple measure of controlling intensity that has been utilised within the studies analysed [16, 26, 27, 29, 30, 34, 36]. In addition, using Rate of Perceived Exertion (RPE) [37] to prescribe exercise intensity is considered appropriate for older adults [65, 66]. However, in many cases, the number of sets, repetitions or recovery periods was missing from the methodology, preventing the protocols being repeated. Only one study [30] explained the control of the intensity of the flexibility whereby the involved muscles were contracting for 60–90 s during stretching.

Intensity should be controlled like with land exercise but with consideration of the principles of hydrodynamics and the forces that influence aquatic exercise to improve physiological responses in older adults [67]. For example, in one study [17], three different types of training were compared over ten weeks: land training with machines (*n* = 14), training with elastic bands (*n* = 21) and water resistance training (*n* = 17) following the same protocol training but adapted to the environment. The three groups obtained significant differences (*p* < 0.05) in flexion of arms (push up), squats, crunches and in the fat-free mass. It is shown that controlled resistance training in the aquatic environment can be as beneficial for the improvement of physical condition and body composition as land training when the protocol is adapted to the specific nature of the environment. In this way, a table of the intervention of the studies is designed, which will serve for clinical protocols and researchers to know the variables of exercise prescription of each of the articles analysed (Table 3).

### Methodology quality

Generally, the methodological quality of the studies included was low. The mean PEDro score for all fourteen studies was 5.5/10 (Table 4). A score of seven (or over) is considered high quality [68], and therefore only on studies in this review had a high quality methodology [33]. Thirteen studies were considered ‘low quality’ when using a threshold of six or less out of ten [16, 24–32, 34–36]. Due to the nature some interventions, it was difficult to blind subjects, therapists and assessors and therefore consistently low scoring items on the scale were: three, five, six and seven.

**Table 4:** Grade of evidence PEDro score

Study	PEDro Score out of 11	Item											Sample size
		1	2	3	4	5	6	7	8	9	10	11	
Silva et al., 2018 [31]	6/10	•	•		•			•		•	•	•	41
Reichert et al., 2018 [16]	6/10	•	•		•			•		•	•	•	36
Seyedjafari et al., 2017 [32]	4/10	•			•					•	•	•	30
Bento et al., 2015 [34]	5/10	•	•		•					•	•	•	36
Kim y O’sullivan, 2013 [28]	5/10	•	•		•					•	•	•	15
Sanders et al., 2013 [36]	5/10	•			•				•	•	•	•	60
Bergamin et al., 2013 [30]	6/10	•	•		•				•	•	•	•	53
Elbar et al., 2013 [33]	8/10	•	•	•	•			•	•	•	•	•	34
Javaheri et al., 2012 [24]	6/10	•	•		•				•	•	•	•	28
Alikhajeh et al., 2012 [35]	4/10	•							•	•	•	•	28
Bento et al., 2012 [29]	5/10	•	•		•					•	•	•	38
Walia y Shefali, 2012 [28]	6/10	•	•		•				•	•	•	•	60
Katsura et al., 2010 [27]	5/10	•	•						•	•	•	•	20
Tsourlou et al., 2006 [25]	6/10	•	•		•				•	•	•	•	22

**•** = Met this criterion OF PEDro Scale

It is difficult to advocate the use of one study over another due to a lack of repeated designs. This makes it difficult to establish a “fixed criteria” in the development of training programmes in the aquatic environment in order to reduce the risk of falls in the elderly.

Although the studies mention the reduction of fall risk with the results obtained, they do not present correlations between variables of strength, balance or flexibility that could suggest a strong clinical relationship. Well-written methodologies should express all relevant information on the training design (volume, intensity, frequency, recovery and type of exercise) in a clear and replicable way. They should also present correlations between falls and physical training variables such as agility, balance, strength and flexibility.

### Study limitations

There is some evidence to support using aquatic exercise for improving predisposing, modifiable risk factors of falls. However, the quality of this evidence is low, and many interventions are poorly described. The lack of consistency between study methodologies makes intervention comparison difficult. In addition, the trial findings did not find a statistically significant relationship between training variables and falls. Resistance equipment is not included to increase exercise intensity, and few studies highlight the importance of execution speed during the aquatic exercises. Regarding the types of exercise, most of them refer to general exercises of upper and lower limbs; however, the exercises should be described in detail so that professionals can achieve the desired results. Regarding the flexibility variable, it is included during the programs directed to older adults but it is not discussed as a relevant variable. Flexibility is not treated in a specific way, obviating the importance of the pelvic musculature in the disturbance of the gait and therefore of the risk of falling. Researchers should avoid describing a superficial and uncritical methodology in order to design specific and valid aquatic programs for reducing the most modifiable predisposing risk factors of falls within the elderly.

## Conclusions

There is some evidence to support using aquatic exercise for improving predisposing, modifiable risk factors of falls. However, the quality of this evidence is low, and many interventions are not fully described. The lack of consistency between study methodologies makes intervention comparison difficult. In addition, the trial findings did not find a statistically significant relationship between training variables and falls.

Despite the inconsistent and lacking results, it should be noted that there were no detrimental or counterproductive effects found within the search results following a physical aquatic exercise programme. With increased consistency in the design of evidence-based training programmes, the aquatic environment could become widespread and effective for helping to prevent the risk of falls in the elderly. Further research is needed to create an evidence-based, replicable protocol for aquatic training with a specific aim of improving the commonly reported predisposing risk factors of falls.

## Abbreviations

ACSM: American College of Sports Medicine
ADL: Activity of Daily Living
PRISMA: Preferred Reporting Items for Systematic Reviews and Meta-Analyses
